# Cecal Bascule in a Cardiac Surgical Patient

**DOI:** 10.7759/cureus.104565

**Published:** 2026-03-02

**Authors:** Victoria E McGuirt, Scott R Coleman, Rohesh J Fernando

**Affiliations:** 1 Anesthesiology, Wake Forest University School of Medicine, Winston-Salem, USA

**Keywords:** bowel, cardiac surgery, cecal bascule, nausea, volvulus

## Abstract

A cecal bascule is a specific type of volvulus that confers a high degree of mortality if it is not addressed. Timely and proper diagnosis is critical, as it generally requires surgical correction. Given the rarity of this phenomenon, it is important for clinicians to be aware of this complication to facilitate treatment. In this case report, we discuss the diagnosis and management of a patient who experienced a cecal bascule postoperatively after cardiac surgery. Prompt diagnosis allowed the patient to obtain appropriate treatment, and he was ultimately discharged after a positive outcome.

## Introduction

Cecal bascule, a type of cecal volvulus, was initially described by Treves in 1899 and was further described by Weinstein, both clinically and radiographically, in 1938 [1]. It is a rare bowel abnormality, with an incidence of only 2.8-7.1 people per million per year [2]. This complication makes up 1-2% of all bowel obstructions and 5-20% of all cecal volvulus cases [2]. Failure to recognize and treat cecal bascule may result in a mortality rate of up to 50%, with nonoperative management of the condition having a 95% failure rate [3]. Overall, it is a rare and deadly manifestation, and it has been reported very few times in association with cardiac surgery. Anesthesiologists play a crucial role in the perioperative management of patients with significant congenital or acquired anatomical anomalies, particularly in the intensive care unit (ICU) postoperatively. Given that they often manage the recovery of these patients, understanding the unique anatomical challenges becomes imperative [4]. We present a case in which a patient who underwent coronary artery bypass graft (CABG) surgery and mitral valve replacement unexpectedly developed a cecal bascule in the postoperative period. The patient provided written consent for publication of this case report. The CARE (CAse REports) checklist, which is an internationally accepted structured framework used to improve the accuracy and transparency of case reports, was used in preparation of this manuscript [5].

## Case presentation

**Figure 1:**
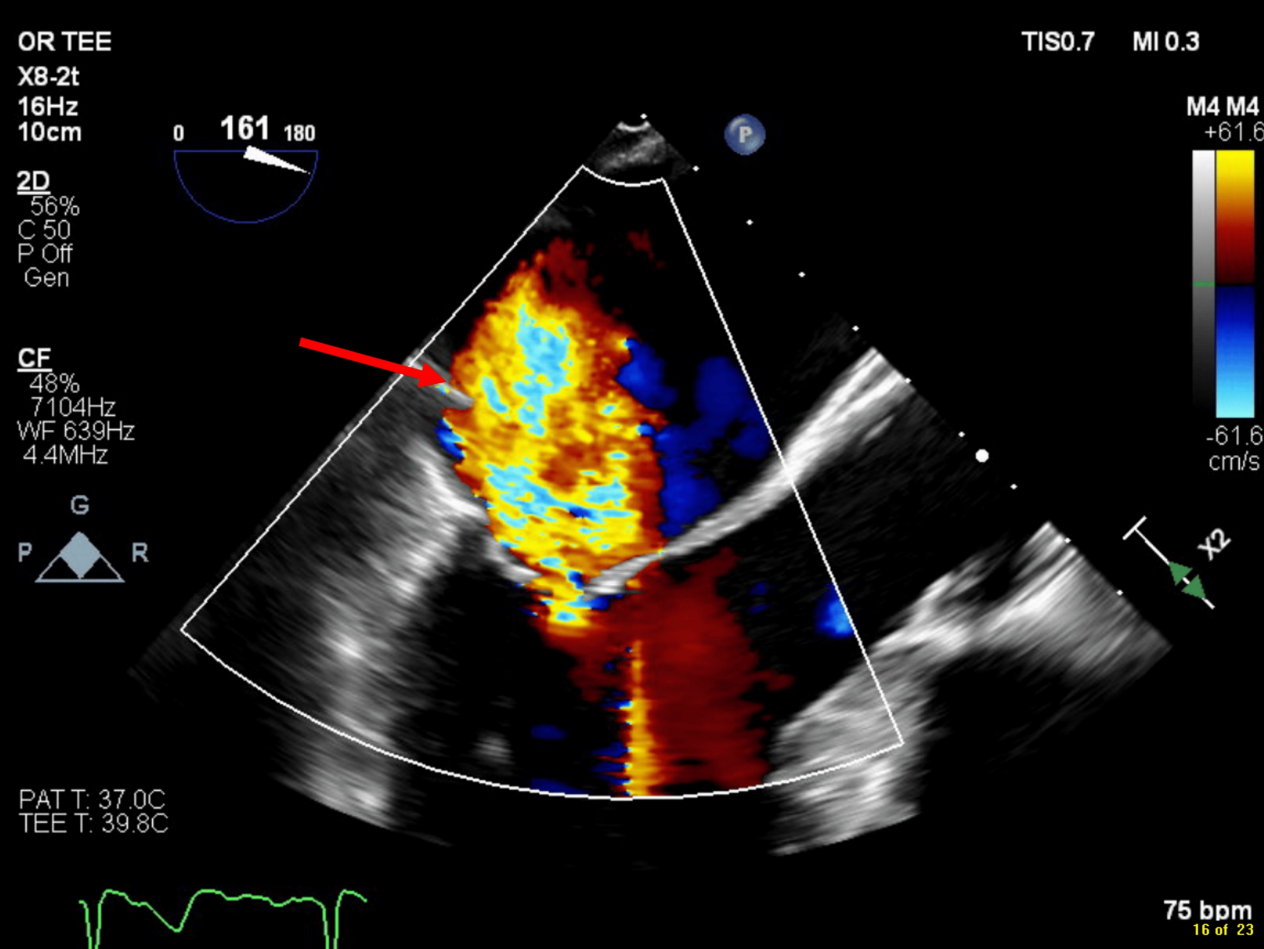
Central component of mitral regurgitation jet. Midesophageal long-axis view obtained with transesophageal echocardiography showing the central component of the mitral regurgitation jet (arrow).

**Figure 2:**
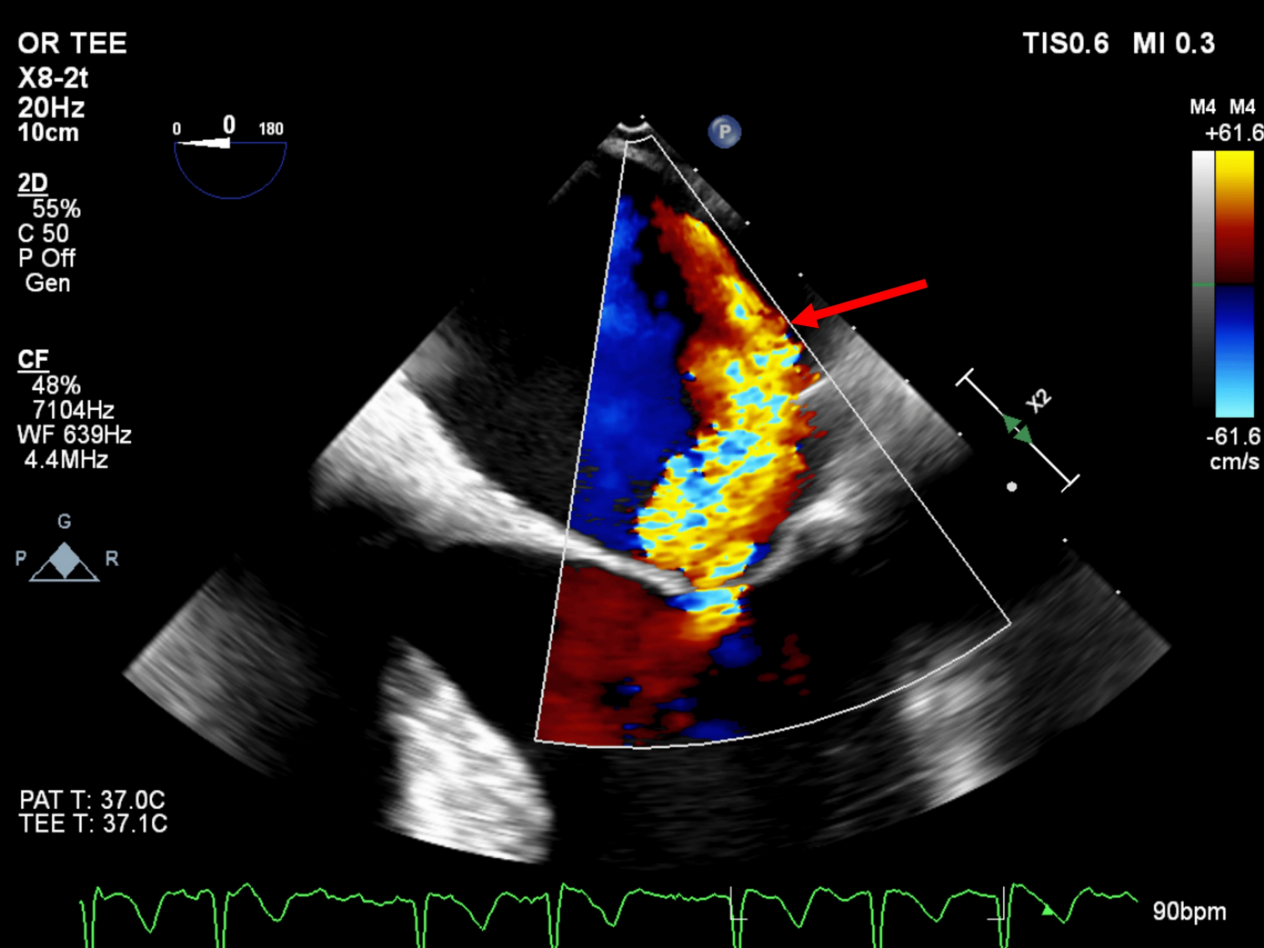
Eccentric component of mitral regurgitation jet. Midesophageal 4-chamber view obtained with transesophageal echocardiography showing the eccentric component of the mitral regurgitation jet (arrow) with associated Coanda effect. The Coanda effect refers to when an eccentric jet is drawn towards the chamber wall and appears to cling to it (in this case, the left atrial wall). These jets are prone to underestimation of severity by jet area using color flow Doppler [6].

A 62-year-old man with past medical history significant for heart failure with preserved ejection fraction, coronary artery disease, paroxysmal atrial fibrillation, obesity, and ventricular tachycardia was scheduled for CABG as a same-day admission case. His preoperative transthoracic echocardiogram showed normal biventricular function and mild to moderate mitral regurgitation. On the day of surgery, the patient underwent general endotracheal anesthesia with standard monitors, as well as arterial, central, and pulmonary artery catheters. Intraoperatively, transesophageal echocardiography revealed a mitral regurgitation jet with central (Figure 1) and eccentric, posteriorly directed (Figure 2) components. Overall, this was consistent with severe mitral regurgitation, which was worse than previously thought. After discussion with the surgeon, the decision was made to also perform mitral valve replacement. The patient received three bypass grafts (left internal mammary artery to left anterior descending artery, greater saphenous vein graft from the ascending aorta to the posterior descending artery, and greater saphenous vein graft from the ascending aorta to the first obtuse marginal artery), mitral valve replacement with a bioprosthesis (29mm Mitris Resilia), and left atrial appendage occlusion. The total cardiopulmonary bypass time was 180 min, with an aortic cross-clamp time of 110 min. Separation from cardiopulmonary bypass required support with infusions of epinephrine (5mcg/min), phenylephrine (25 mcg/min), inhaled epoprostenol (50ng/kg/min), as well as an intra-aortic balloon pump. Analgesia for the case was achieved with 10 mg methadone and 200 mcg fentanyl intraoperatively. He received a total of 1.75 L of crystalloid and 683 mL of cell salvage. The remainder of the surgery was unremarkable, and he was transferred to the cardiovascular intensive care unit, where he continued to receive support with infusions of epinephrine (5mcg/min), phenylephrine (0-50mcg/min) and inhaled epoprostenol (50ng/kg/min).

**Figure 3:**
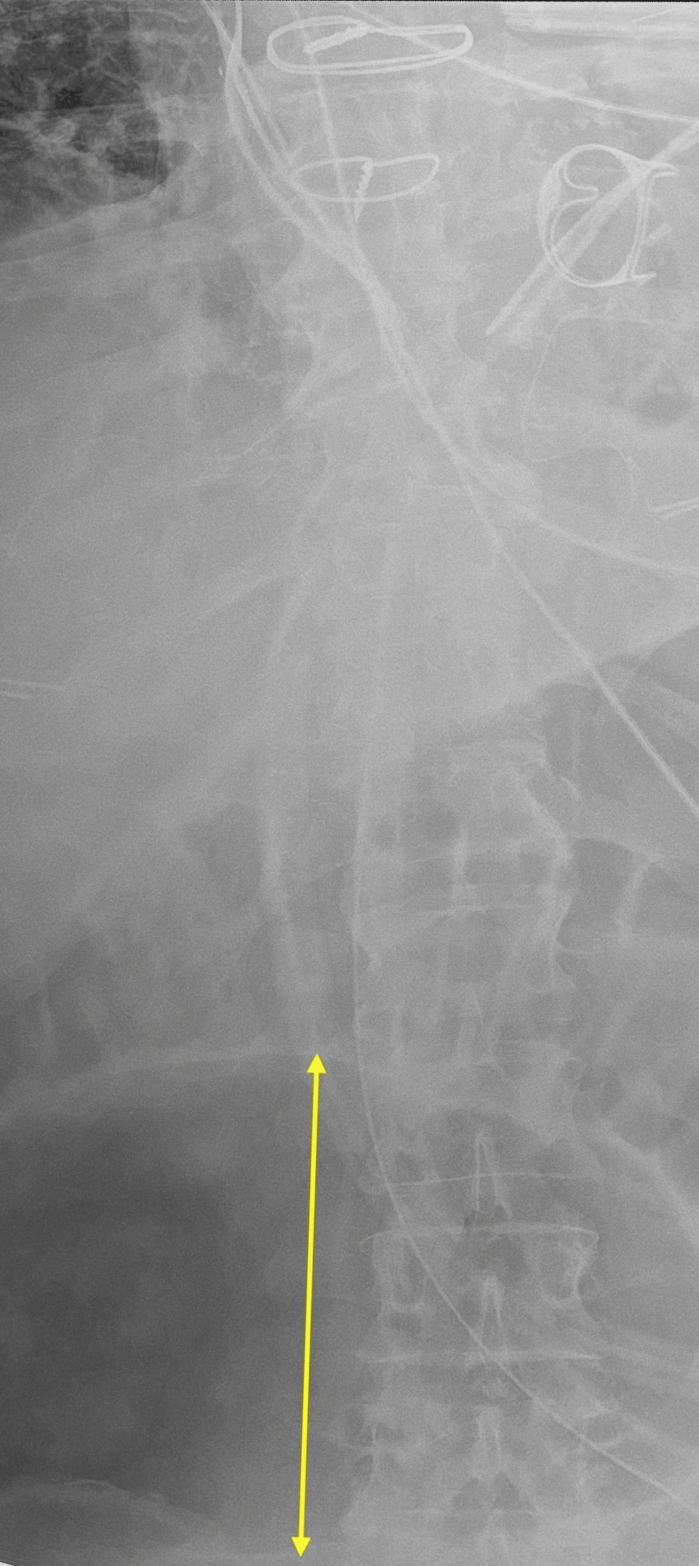
Abdominal radiograph An enlarged cecum is shown, measuring 12 cm (arrow), concerning for cecal bascule.

**Figure 4:**
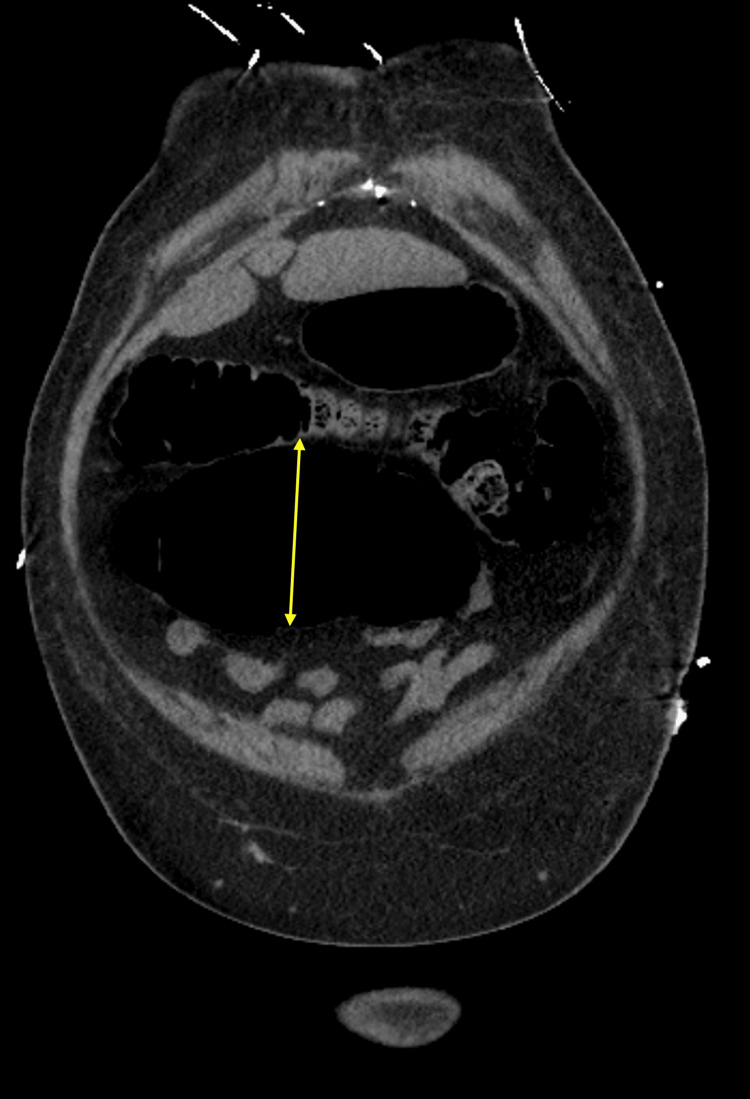
Computed tomography image Coronal image from a computed tomography scan of the abdomen showing a dilated cecum (9.3 cm, arrow) with an anteromedial orientation, concerning for cecal bascule.

Postoperatively, a milrinone infusion was added for additional inotropic support (initially 0.125mcg/kg/min and increased to 0.25mcg/kg/min). He was extubated on postoperative day (POD) one and given a regular diet, which he tolerated without complication. Analgesia was achieved with either oral oxycodone or hydromorphone, intravenous ketorolac, oral acetaminophen, and intravenous fentanyl for breakthrough pain. On POD 2, the left ventricular function returned to normal without any inotropic support, and the intra-aortic balloon pump was removed. On POD 3, the patient complained of significant abdominal distention and nausea without emesis. Physical exam demonstrated abdominal tenderness. An abdominal radiograph was obtained, which showed an enlarged cecum of up to 12 cm wide (Figure 3). An abdominal CT scan was subsequently ordered, which demonstrated a mildly distended cecum (9.3 cm) with anteromedial orientation, suggestive of cecal bascule (Figure 4). A nasogastric tube was placed with 500 mL of bilious output. The emergency general surgery service was consulted, who took the patient emergently to the operating room for exploratory laparotomy. A rapid sequence induction was performed due to elevated aspiration risk. Surgical findings included a turned-over cecum that was extremely dilated, consistent with cecal bascule, with no evidence of ischemia or perforation. He underwent open right hemicolectomy and was extubated at the conclusion of the procedure. On POD 4, the patient removed his own nasogastric tube due to discomfort. His diet was advanced to clear liquids on POD 5, full liquids on POD 7, and a regular diet on POD 8. He was ultimately discharged on POD 11 after implantation of an internal cardioverter-defibrillator. Overall, the patient seemed satisfied with his hospital treatment, but described that it was stressful to have so many people talk to him while he was in the hospital about issues that he perceived to be unimportant.

## Discussion

The development of a cecal bascule is a very rare condition [2]. To our knowledge, there are only two other case reports in cardiac surgery that highlight this complication [3,7]. The cecum is the area of the intestinal tract that connects the small intestine to the large intestine. It is susceptible to folding, otherwise known as a volvulus. There are three types of cecal volvulus, which are: axial torsion (type 1), loop (type 2), and cecal bascule (type 3) [2]. The first two types comprise about 80% of these cases, while type 3 accounts for 5-20% of cases [2]. A cecal bascule is caused by an upward folding of the cecum and movement to the upper right quadrant of the abdomen, and typically occurs in patients aged 40-70 years and more often in males [2]. Surgical management is optimal, with right hemicolectomy being the ideal treatment given the rare incidence of recurrence. Although there are other surgical options, such as a cecostomy tube and cecopexy, these are typically used in unstable patients, given that the outcomes are not as good [3].

Risk factors for cecal bascule are difficult to elucidate given the rarity of this condition, but include high fiber intake, previous abdominal surgery, chronic constipation, ileus, and distal colonic obstruction [3]. There have also been links between previous pregnancy or postpartum abdomen and cecal bascule [8]. In addition, colonic pseudo-obstruction has been associated with cecal bascule [9]. To our knowledge, the patient did not have any of these specific risk factors.

Clinically, patients with cecal bascule may present with signs such as abdominal pain and discomfort, abdominal distention, diarrhea, and postoperative nausea and vomiting (PONV). Unfortunately, PONV after cardiac surgery is not uncommon. Some studies report an incidence ranging from 42-71% in the cardiac surgical patients, while general surgical rates are roughly 20-30% [10]. Since many of the presenting signs and symptoms of cecal bascule, such as PONV, are not specific to this anomaly, it can make cecal bascule more challenging to diagnose. Additionally, symptoms may be quickly attributed to other conditions that are more common, such as ileus, without consideration of less common pathologies.

Providers should therefore find ways to differentiate cecal bascule from other abdominal conditions. In addition to the symptoms mentioned above, it can also present with hemodynamic instability and confusion in severe cases [11]. Imaging can significantly aid in the diagnosis. While a plain abdominal radiographic film can be helpful, the findings of a dilated cecum and “inverted teardrop” appearance are nonspecific [3]. Therefore, computed tomography (CT) is commonly used and can be helpful in clarifying the diagnosis [3]. When a cecal bascule is present, a gas-filled cecum pushed anteriorly to the ascending colon will appear with a fold [3]. CT scans are diagnostic in 90% of cases and can show the cecal bascule, as well as other complications, such as pneumatosis intestinalis, pneumoperitoneum and mesenteric stranding [3].

Ileus is a common abdominal complication that can occur in cardiac surgical patients, and it should be considered in the differential as it can also influence patient morbidity and length of stay. There are three major types of postoperative ileus, which are pan-intestinal, upper gastrointestinal (GI), and lower GI [12]. Pan-intestinal ileus presents as large or small bowel dysmotility, inability to have bowel movements, vomiting, distention, pain, and decreased oral intake [12]. Upper GI ileus presents as small bowel dysmotility with small amounts of stool from partial GI function, nausea, vomiting, and sometimes distention. Lower GI ileus presents as large bowel dysmotility with no stool passing, but patients may not exhibit symptoms such as nausea or vomiting if they are only taking a liquid diet. Clinical management includes inserting a nasogastric tube, correcting electrolyte abnormalities, and intravenous fluid administration [12]. Other potential diagnoses to exclude are acute cholecystitis, acute diverticulitis, acute pancreatitis, peptic ulcers, and GI bleeding [13]. Bowel ischemia is a potentially deadly complication that can result from ileus and is also important to consider when a patient has had an intra-aortic balloon pump [13].

Anesthetic considerations for this patient were focused on the concern for aspiration. In this case, a nasogastric tube allowed for decompression of the stomach prior to anesthetic induction. Furthermore, a rapid sequence induction was performed to secure the airway in a timely fashion and avoid mask ventilation. Fortunately, the patient’s cardiac function had sufficiently recovered that he tolerated induction well from a hemodynamic standpoint. In cardiac surgical patients with depressed ventricular function, induction may require additional inotropic support and/or vasopressors, and an arterial line may be beneficial.

A strength of this report is that the patient was in the hospital when the cecal bascule occurred, so the details and the timing of the postoperative events were carefully documented. In addition, imaging was readily available for further evaluation after symptoms occurred, and surgical exploration confirmed the diagnosis. A limitation is that this is a single report from a single center, which limits the generalizability.

## Conclusions

In summary, we present a case of a patient with a cecal bascule. Although rare, this can occur after cardiac surgery and is life-threatening. Medical providers should consider this in their differential diagnosis, particularly when imaging is concerning. While symptoms may overlap with other common postoperative complications such as ileus, imaging such as computed tomography can be particularly useful to differentiate cecal bascule from other abnormalities. Overall, despite the unexpected occurrence of this serious complication, this patient was able to get corrective surgery in a timely fashion and was ultimately discharged with a positive outcome.
